# Recurrence outcome of lymph node ratio in gastric cancer after underwent curative resection: A retrospective cohort study

**DOI:** 10.1016/j.amsu.2020.04.002

**Published:** 2020-04-19

**Authors:** Chairat Supsamutchai, Chumpon Wilasrusmee, Jakrapan Jirasiritham, Teerawut Rakchob, Songpol Phosuwan, Tanet Chatmongkonwat, Pattawia Choikrua, Tharin Thampongsa

**Affiliations:** Department of Surgery, Faculty of Medicine Ramathibodi Hospital, Mahidol University, Thailand

**Keywords:** Gastric cancer, Lymph nodes ratio, D2 gastrectomy, Curative, Lymphadenectomy

## Abstract

**Introduction:**

D2 dissection has been regarded as the standard procedure for locally advanced gastric cancer (GC). Number of lymph nodes (LN) harvested is an important factor for accurate staging. The number of LN retrieved and the metastasis LN status are also important factors to determine the prognosis. This study aims to evaluate whether lymph node ratio (LNR) could be a prognostic indicator of GC patients following curative resection.

**Patients and methods:**

Single center retrospective cohort study of GC patients underwent curative resection from January 1995 to December 2016 was conducted. The patients were categorized into 3 groups based on LNR (0.00–0.35, >0.35–0.75, and >0.75–1.00) and 2 groups based on number of LN retrieved (<15 and ≥ 15). Kaplan-Meier method was used to estimate recurrence-free survival. Cox-regression were used to determine the association between LNR/other factors and the disease recurrence.

**Results:**

One-hundred fifty-three patients were included in analysis. Univariate analysis showed that LNR >0.35, pathologic LN stages (pN) 2–3, higher number of LN metastasis, and TNM stage III were significantly recurrence risk factors. After adjusting for several covariates, LNR >0.35 still was significant predictor (adjusted HR [95%CI], 8.53 [1.97, 36.86]; *p* = 0.004) while number of LN retrieved or number of metastasis LN were not.

**Conclusion:**

LNR could be a strong indicator for the recurrence of GC after curative resection while the number of LN retrieved or metastasis did not predict the recurrence. Future studies, such as prospective studies, are needed to confirm and identify the optimum LNR cut-off.

## Highlights

• A study to assess the prognostic value of lymph node ratio (LNR) for gastric cancer patients after curative gastrectomy.
• LNR and number of metastatic LNs were independent prognostic factors of gastric cancer and other malignant neoplasm.
• In the present study, LNR was a strong predictor for the local recurrence while the number of metastatic LNs was not.
• LNR >0.35 indicates poor prognosis after curative gastrectomy.

## 1 Introduction

Gastric cancer was the fifth most common cancer related death in the world [1]. There are several factors associated with the prognosis of disease. One of them was the presence of lymph node metastasis after curative surgery [[2], [3], [4], [5]]. The lymph node ratio between lymph node metastasis and total lymph node retrieved has been proposed as a new prognosis factor from recent studies.

There are two major worldwide guidelines for classifying the status of lymph node metastasis in gastric cancer, i.e. the Union for International Cancer Control and American Joint Commission for Cancer (UICC/AJCC) and Japanese Gastric Carcinoma Association (JGCA). The Japanese gastric cancer guidelines used anatomical location and the type of lymphadenectomy [6,7]. The D2 gastrectomy is a standard procedure and has been recommended as optimal treatment for early gastric cancer in Japan [7]. While UICC/AJCC staging system considered only the number of lymph node metastasis which the total lymph node dissection should be more than 15 lymph nodes [8]. Hence, the number of lymph nodes harvested during the gastric resection is an important factor to determine the accuracy for cancer staging.

Regardless of the guidelines, the lymph node ratio (LNR) that uses information of the number and pathological results of lymph node after surgery to determine cancer survival, could be applied in clinical. Therefore, this study aims to evaluate the association between the number of LN that retrieved from the curative resection, number of metastatic lymph nodes, and the lymph node ratio (LNR) and the recurrence rate after curative resection of gastric cancer.

## 2 Patients and methods

### 2.1 Study design, setting, and patients

This was a retrospective cohort study of the gastric cancer patients underwent curative surgery resection from January 1, 1995 to December 31, 2016 in Ramathibodi Hospital, Bangkok, Thailand. The ethical approval was obtained prior to commencing study. The study was conducted in accordance with the Declaration of Helsinki. The study was registered at Clinicaltrials.gov (NCT03778710).

The patients who underwent curative surgery resection were included in the review and study analysis if the patient was ≥18 years old at the time of surgery, the gastric cancer diagnosis confirmed by histology, did not previously receive neoadjuvant therapy and no distant metastasis at the time of surgery from an imaging study, such as ultrasonography, computer tomography (CT) or magnetic resonance imaging (MRI). The patients who presented with distant metastasis during surgery or incompletion of tumor-node-metastasis (TNM) parameters for TNM staging were excluded.

### 2.2 Medical and surgical interventions

The gastrectomy was performed with curative intent and D2 lymphadenectomy according to Japanese guideline for gastric cancer [6]. After the surgery, the patients received adjuvant therapy following the standard regimen.

### 2.3 Study data collection and outcome of interest

The patients’ medical records and pathological reports were reviewed to obtain patient data, including age at the time of surgery, sex (male or female), degree of tumor differentiation (differentiated or undifferentiated), pathologic stage of disease; pT, pN and pM (tumor-node-metastasis) parameters according to the TNM classification of AJCC. The stages of disease from pathological reports were read and confirmed by pathologist.

The LNR was determined by number of positive lymph nodes and number of total lymph nodes retrieved from the curative surgery. LNRs were divided into 3 groups at approximately 75^th^ and 95^th^ percentiles, i.e. 0.00–0.35, >0.35–0.75 and >0.75–1.00. Based on the UICC and AJCC since 1997, at least 15 lymph nodes should be examined to ensure complete resection and adequate staging [9]. We therefore divided the patients into 2 groups based on total number of lymph node retrieval, i.e. less than 15 and equal to or more than 15, to assess the differences in clinicopathological characteristics. The staging of tumors was according to AJCC classification. The disease recurrence after the curative resection were retrieved from the patient medical records.

### 2.4 Statistical analysis

The data from all gastric cancer patients who underwent the curative surgery resection during the planned study period and met the eligibility criteria were included in the study analysis. The data was analyzed by STATA version 14.0. Chi-square (or Fisher's exact test) and *t*-test (or median test), as appropriate, were used to identify the differences in the clinicopathologic characteristics between 2 groups. Chi-square (or Fisher's exact test) and ANOVA (or median test) were used to identify the differences between LNR groups. The Kaplan Meier method was used to estimate the probability of the recurrent free survival (RSF). Log rank test was used to test the difference in recurrence free survival (RFS) among groups. Uni- and multivariate Cox proportional hazard models were used to identify predictors of the disease recurrence. The *p*-value < 0.05 was considered to represent a statistically significant difference. The results were presented in line with the STROCSS criteria[10].

## 3 Results

### 3.1 Patients’ clinicopathological characteristics

There was a total of 158 patients of gastric cancer who underwent curative gastric resection. Five patients (3.16%) were excluded due to incompletion of pT or pM parameters for TNM staging. One hundred fifty-three (153) patients were included in the study analysis. The follow-up time of this study was approximately 2 years (median (IQR) 702 (383, 1,193) days) and the mean age (SD) was 58.90 (12.64) years. Table 1 presents the clinicopathologic characteristics of the patients by total lymph node retrieval less than 15 nodes and equal or more than 15 nodes and Table 2 presents the characteristics by the LNR ranges.

**Table 1:** Clinicopathologic characteristics of patients by number of total lymph node retrieval.

	Number of total LN retrieval (nodes)		*p*-value
	<15	≥15	
	(n = 15)	(n = 138)	
Age (yrs.), mean (SD)	62.07 (11.82)	58.56 (12.72)	0.309
Gender			
Male	10 (66.67)	67 (48.55)	0.183
Female	5 (33.33)	71 (51.45)	
Histology (n = 142 **)			
Differentiated	5 (38.46)	42 (32.56)	0.759
Undifferentiated	8 (61.54)	87 (67.44)	
Pathological stage; n (%)			
Tumor (T)			
pT1	2 (13.33)	6 (4.35)	0.141
pT2	1 (6.67)	21 (15.22)	
pT3	7 (46.67)	88 (63.77)	
pT4	5 (33.33)	23 (16.67)	
Lymph nodes (N)			
pN0	4 (26.67)	30 (21.74)	0.165
pN1	5 (33.33)	49 (35.51)	
pN2	5 (33.33)	21 (15.22)	
pN3	1 (6.67)	38 (27.54)	
Metastasis (M)			
pM0	12 (80)	134 (97.10)	0.021*
pM1	3 (20)	4 (2.90)	
TNM Stage			
I	2 (13.33)	14 (10.14)	0.793
II	5 (33.33)	58 (42.03)	
III	8 (53.33)	66 (47.83)	
Number of LN; median (IQR)			
Number of LN metastasis	1 (0, 5)	4 (1, 12)	0.042*
Number of total LN retrieval	9 (4, 12)	39 (30, 51)	<0.001*
LNR	0.33 (0, 0.56)	0.098 (0.025, 0.308)	0.186

*indicates statistically significant difference (p < 0.05).

** Numbers may not add to the total because of missing data.

Abrarivations: IQR, interquartile range; LN, lymph node; LNR, lymph node ratio; pT/pN/pM, pathologic TNM staging; SD; standard deviation.

**Table 2:** Clinicopathologic characteristics of patients by lymph node ratio (LNR) groups.

	LNR			p-value
	0.00–0.35	>0.35–0.75	>0.75–1.00	
	n = 116	n = 29	n = 8	
Age (yrs.), mean (SD)	58.33 (12.63)	58.34 (12.06)	65.50 (14.54)	0.296
Gender				
Male	61 (52.59)	13 (44.83)	3 (37.50)	0.570
Female	55 (47.41)	16 (55.17)	5 (62.50)	
Histology (n = 142 **)				
Differentiated	40 (37.74)	7 (25.00)	0	0.056
Undifferentiated	66 (62.26)	21 (75.00)	8 (100)	
Pathologic stage, n (%)				
Tumor (T)				
pT1	8 (6.90)	0	0	0.303
pT2	20 (17.24)	2 (6.9)	0	
pT3	69 (59.48)	21 (72.41)	5 (62.50)	
pT4	19 (16.38)	6 (20.69)	3 (37.50)	
Lymph nodes (N)				
pN0	34 (29.31)	0	0	<0.001*
pN1	52 (44.83)	0	2 (25)	
pN2	18 (15.52)	7 (24.14)	1 (12.50)	
pN3	12 (10.34)	22 (75.86)	5 (62.50)	
Metastasis (M)				
pM0	111 (95.69)	29 (100)	6 (75)	0.034*
pM1	5 (4.31)	0	2 (25)	
TNM Stage				
Stage I	16 (13.79)	0	0	<0.001*
Stage II	63 (54.31)	0	0	
Stage III	37 (31.90)	29 (100)	8 (100)	
Number of LN; median (IQR)				
Number of LN metastasis	2 (0, 6)	17 (11, 24)	15.5 (6, 32.2)	<0.001*
Number of total LN retrieval	38 (28, 50.5)	39 (20, 52)	16 (6.5, 38)	0.310
LNR, median (IQR)	0.06 (0, 0.17)	0.50 (0.43, 0.52)	0.92 (0.88, 1)	<0.001*

*Indicates statistically significant difference (p < 0.05).

Abbreviation: IQR, interquartile range; LN, lymph node; LNR, lymph node ratio; pT/pN/pM, pathologic TNM staging; SD; standard deviation.

Median LNR (IQR) was 0.11 (0.03, 0.34). There were no significant differences in node stages, TNM stages and LNR between the patients with total lymph node retrieval less than 15 nodes and equal or more than 15 nodes. However, all patients with LNR >0.35 were pathologic TNM stage III gastric cancer while approximately one third approximately one-third of the patients with LNR ≤0.35 were pathologic stage III gastric cancer (*p*-value <0.001).

### 3.2 LNR and recurrence risks

Within study observation period, 28 of 153 patients experienced local recurrence.

The RFS rates (95% CI) were 94.14% (88.61–97.03%), 85.95% (77.87–91.24%), 73.07% (62.51–81.09%), 71.48% (60.64–79.82%), and 69.17 (57.61%, 78.16%) at 1, 2, 3, 4 and 5-years, respectively. Seven patients had metastasis. The most common site of metastasis was peritoneal metastasis (4 of 7 patients). One patient had liver metastasis and 2 patients had lung metastasis.

**Fig. 1:**
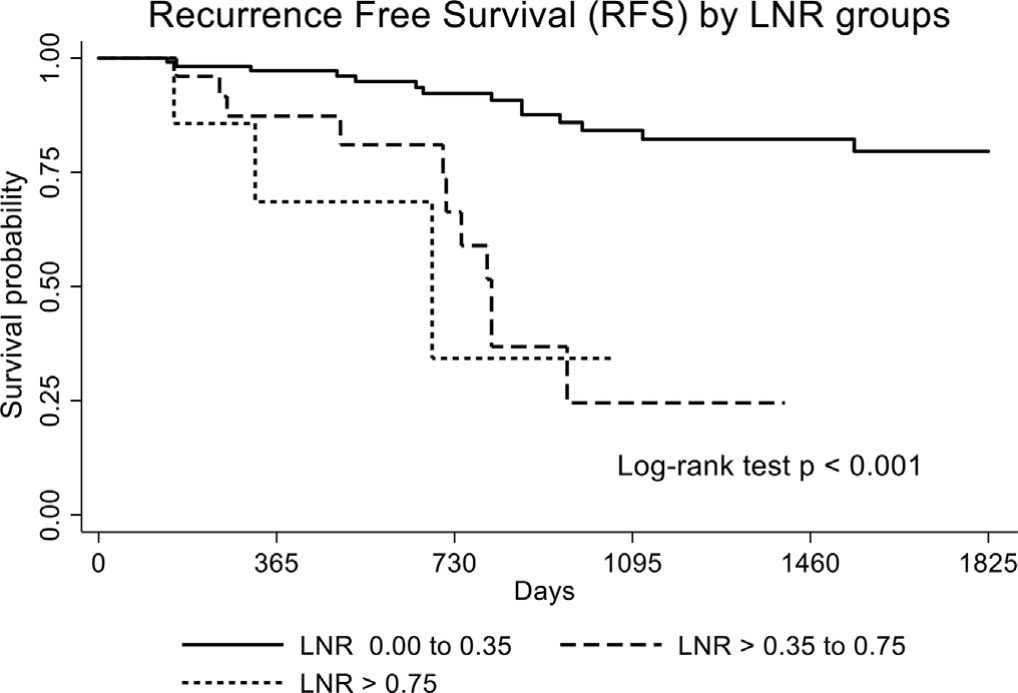
Local Recurrence Survival by Lymph Node Ratio Groups.

There were significant differences among the LNR groups (0.00–0.35, >0.35–0.75 and > 0.75) in terms of local recurrence-free survival (log-rank test *p*-value < 0.001). The RFS rates 2 years after the operation were 92.30%, 66.30%, and 34.29% for LNR 0.00–0.35, >0.35–0.75 and > 0.75, respectively (Fig. 1). Univariate Cox regression analysis showed that the patients with LNR >0.35–0.75 and > 0.75 had significantly higher rates of local recurrence when compared those with LNR <0.35 (HR (95% CI), 6.35 (2.79–14.49) and 7.89 (2.19, 28.32), respectively. In addition to LNR or LNR groups, univariate analysis also showed that the patients with pN2, pN3, higher number of lymph node metastasis, and gastric cancer stage III were significantly associated with a higher local recurrence risk (Table 3) while total number of lymph node retrieval ≥ 15 nodes had significant lower risk of recurrence (HR (95% CI): 0.36 (0.14, 0.95)). Age, degree of tumor differentiation, and gender did not predict the disease recurrence.

**Table 3:** Univariate and multivariate Cox regression analysis of local recurrence risk (n = 153).

	N	Recurrence	Univariate		Multivariate	
			HR (95%CI)	p-value	HR (95%CI)	p-value
Age, yr	153	28	1.01 (0.99, 1.04)	0.282		
Gender						
Male	77	11	Reference			
Female	76	17	1.53 (.72, 3.28)	0.269		
Histology (n = 142)						
Differentiated	47	5	Reference			
Undifferentiated	95	20	2.08 (0.78, 5.54)	0.144		
pN**						
pN0	34	2	Reference		Reference	
pN1	54	10	2.71 (0.59, 12.41)	0.198	2.23 (0.46, 10.91)	0.321
pN2	26	6	5.09 (1.03, 25.29)	0.046*	0.82 (0.08, 8.05)	0.862
pN3	39	10	7.47 (1.62, 34.49)	0.010*	0.64 (0.05, 8.96)	0.744
Number (N) of LN						
N of LN metastasis**	153	28	1.05 (1.01, 1.09)	0.007*	0.98 (0.91, 1.06)	0.677
N of total LN retrieval	153	28	0.98 (0.96, 1.00)	0.062		
N of total LN retrieval**						
< 15 nodes	15	5	Reference		Reference	
≥ 15 nodes	138	23	0.36 (0.14, 0.95)	0.040*	0.49 (0.15, 1.6)	0.239
LN ratio (LNR) group						
0.00–0.35	116	14	Reference			
> 0.35–0.75	29	11	6.36 (2.79, 14.49)	<0.001*		
>0.75–1.00	8	3	7.89 (2.2, 28.32)	0.002*		
LNR groups**						
0.00–0.35	116	14	Reference		Reference	
> 0.35–1.00	37	14	6.63 (3.04, 14.43)	<0.001*	8.53 (1.97, 36.86)	0.004*
TNM stage**						
Stage I −II	79	9	Reference		Reference	
Stage III	74	19	4.16 (1.84, 9.39)	0.001*	2.99 (0.75, 11.96)	0.122

Abbreviation: HR, Hazard ratio; LN, lymph node; LNR, lymph node ratio; pT/pN/pM, pathological TNM staging; SD; standard deviation.

* Statistically significant p--value < 0.05.

**Variables included in multivarite Cox proporational hazard model.

After adjusting for several covariates (the variable with *p*-value < 0.10 from the univariate analysis) in the model, LNR > 0.35 still was an only predictor of recurrence in gastric cancer after the curative gastric resection with D2 lymphadenectomy in our study (Adjusted HR (95%CI): 8.53 (1.97, 36.86)) when compared with those in the lower LNR group.

## 4 Discussion

In our present study, there was no difference in cancer stages between the patients with <15 lymph node retrieval and ≥15 lymph node retrieval during the curative gastrectomy. In addition, the total number of lymph node (as continuous) retrieval or the retrieving of <15 or ≥ 15 nodes were not associated with the recurrence free survival in both groups. Besides, there was no significant difference in TNM cancer stage between the lymph node retrieval less than 15 or more groups. In contrast, a previous retrospective study by Biffi et al. [11] concluded that the patients who had ≤15 nodes removed had significantly worse disease-free survival than other patients and suggested more extended LN resection to protect inadequate removal of lymph nodes [11]. However, they did not report the correlation of the LNR.

A recent retrospective study by Hu et al. [12] reported that TNM stage III and LNR were prognostic factors of worse RFS while only LNR was a significant indicator predicting disease-free survival in our study. They also identified and reported that LNR >0.25 could be the most appropriate LNR cut-off value for predicting RFS (HR 2.33, 95% CI 1.33, 4.06) and indicates poor prognosis [12]. This cut-off value was also reported by many researchers for evaluation the prognosis of gastric cancer [13].

In our study, we divided LNRs at 0.35 (approximately 75^th^ percentile). At this cut-off point, the LNR >0.35 still was a strong indicator of worse RFS after adjusting with several covariates. We further performed receiver-operating characteristic (ROC) curve analysis and area under the curve to identify the optimal cut-off values of LNR using our study data and found that, without adjusting for any covariates, the optimal cut point was 0.25 (unadjusted HR 5.54, 95% CI; 2.53, 12.16, *p*-value < 0.001) as same as the previous studies.

Similar to the previous study by Lee et al. [14], they reported that LNR was an independent prognostic factor but the number of metastatic lymph nodes was not. In addition, the study by Alatengbaolide et al. also concluded that the metastatic LNR was an independent prognostic factor regardless of the examined number of lymph nodes [15]. A previous report in western patients also confirmed the role of the LNR as a prognostic factor in western gastric cancer patients treated with D1 lymphadenectomy [16]. However, the lymph node metastasis status was also impact to prognosis in their study. Saito et al. reported that both the number and level of lymph node were useful for evaluating the status of lymph node metastasis [17]. LNR groups in our study (≤0.35 and > 0.35 or ≤ 0.35, 0.35–0.75 and > 0.75) is independent by the number of node retrieved, and therefore the LNR groups could be a useful prognostic indicator in case of conventional lymphadenectomy.

Although, both LNR and the number of lymph node metastasis were used to predict the RFS in many studies. But in the cases that the number of lymph node retrieval was less than 15, the LNR might be an important helpful tool to predict recurrence free survival. Other than LNR and number of lymph node metastasis, there was a study reported that the pN stage was as an important indicators of overall survival [13]. However, our result supports only LNR as prognostic indicator and did not find the association between pN stage or number of metastatic lymph nodes and disease recurrent rate.

Our study has some limitations. By retrospective study design, both known and unknown factors could not be controlled, such as medication given prior to the surgery. This study had small number of patients and 5 patients were excluded due to missing T, N, M data. The study did not consider the histological subtypes or the type of adjuvant therapy in analysis.

In conclusion, the present study results supported and confirmed the promising role of the LNR as a prognostic factor for gastric cancer patients undergoing curative surgery while did not support the number of LN retrieved or LN metastasis as predictor for the disease recurrence. Future studies with more rigorous designs and larger sample sizes, such as prospective cohort studies, are needed to confirm the impact of LNR on prognosis of gastric cancer patients and identify the optimum LNR cut-off in order to be used as a prognostic factor in routine clinical practice.

## Funding

None.

## Provenance and peer review

Not commissioned, externally peer reviewed.

## Declaration of competing interest

None.
